# Suggestions for the Treatment of Eye Wounds

**Published:** 1918-09

**Authors:** 


### SUGGESTIONS FOR THE TREATMENT OF EYE WOUNDS

In the following condensed suggestions for ophthalmic surgeons and other medical officers who have to treat injuries to the eye, the Senior Consultant in ophthalmology has given volumes of sound advice.

*Contusions of Eye Without Rupture.* Guarded prognosis, watch tension especially if traumatic cataract develops. Look for and record any choroidal injury.

*Contusions of Eye With Rupture.* If extensive with great loss of globe contents, enucleate. If less extensive with little loss of contents, suture and cover with conjunctival flaps after freeing wound of any prolapsed tissue.

*Penetrating Wounds.* With foreign body inclusion probable, apply the magnet test. If this shows foreign body to be magnetic remove by magnet, preferably by anterior route if small, and if large through wound of entrance. If unable to remove small foreign body with the magnet at hand, send case to the Base Eye Center. Where foreign body is non-magnetic and not easily removable, follow expectant treatment, as such foreign bodies are often encysted. It should be especially emphasized that penetration of the eye by a foreign body, without a discoverable wound of entrance, is not an uncommon occurrence. All suspicious eyes should therefore be thoroughly investigated for a foreign body by means of the magnet, the ophthalmoscope and by the X-ray when necessary.

*Penetrating Wounds Without Foreign Body Inclusion* should be immediately covered with a conjunctival flap after freely removing prolapsed tissue, then treated as any eye wound. Where an eye is too badly injured to save, or where sympathetic ophthalmia is to be feared and no vision likely to result from conservative methods, the eye should be enucleated. At time of enucleation some material, preferably one of the large glass spheres, should if possible be emplanted in Tenon's capsule. If this cannot be done, it is at least imperative that the four recti muscles should be sutured together. In penetrating injuries of the eye, showing proptosis to a greater or less degree, a thorough perforation of the globe should be suspected.

*Panophthalmitis.* When a penetrating injury results in panophthalmitis it is best to eviscerate and this operation should be the one chosen for a hopelessly injured eye complicated by orbital cellulitis. After enucleation or evisceration artificial eye should be fitted as soon as the socket permits.

*Orbital Injuries.* A small foreign body in the orbit not easily accessible should be left unless cellulitis develops. Larger bodies should be removed, avoiding injury to muscles and nerves. Orbital cellulitis requires free drainage. Perforating wounds of orbits by bullets or other missiles should be treated tentatively. Lagophthalmos from the extreme exophthalmos, often seen in such cases, or the lagophthalmos resulting from facial palsies should have the lids sutured together to protect the eye if there are any signs that the cornea is likely to become involved.

